# Passive fit and time efficiency for prefabricated versus conventionally constructed cobalt chromium CAD\CAM 3-unit implant supported frameworks in free end saddle models: a pilot invitro study

**DOI:** 10.1186/s12903-024-04950-y

**Published:** 2024-10-15

**Authors:** Mohamed El-Sayed Kamel, AlHassan Alaa Eldin Alsayed, Mohamed Amr ElKhashab, Nancy Nader, Iman AbdelWahab Radi

**Affiliations:** 1 Professor of Prosthodontics and Member of Evidence Based Dentistry Centre, Faculty of Dentistry, Cairo University, Vice Dean of School of Dentistry, Badya University, 11 Al Saraya, Al Manial, Giza Governorate, Cairo, Egypt; 2 Professor of Prosthodontics, Faculty of Dentistry, Cairo University , Cairo, Egypt; 3 Vice Dean of School of Dentistry, Badya University, Cairo, Egypt; 4 Master Degree Candidate of Oral Implantology, Faculty of Dentistry, Cairo Univeristy, Cairo, Egypt; 5 Lecturer of Prosthodontics, Faculty of Dentistry, Cairo University, Cairo, Egypt

## Abstract

**Background:**

The passive fit of 3-unit implant supported prefabricated metal screw-retained prosthesis before implant placement might be difficult. Hence, we aim to evaluate the passive fit and time efficiency of CAD/CAM 3-unit implant supported fixed prostheses that were constructed based on virtual versus those based on actual implant positions in Kennedy Class I models.

**Methods:**

A sample of 5 Kennedy class I models with thin wiry ridges were restored by 20 frameworks bilaterally, 10 based on actual (group A) and 10 based on virtual (group V) implant positions. The models were imaged using cone beam computed tomography and scanned using an intraoral scanner. The STL (Standard Tessellation Language files) and the DICOM (Digital Imaging and Communications in Medicine) files were registered on a 3D planning software. A CAD/CAM surgical guide was planned, resin printed and used for installing 6 implants bilaterally. In group V, the framework was designed based on the virtual scan bodies and virtual multi-unit abutments, while in group A intra-oral scanning of the model after attaching the scan bodies was necessary. Frameworks of both groups were milled and tested for passive fit using 8 clinical tests. McNemar and Wilcoxon signed rank tests were used to study the effect of the group on passive fit and time efficiency, respectively. The significance level was set at *P* ≤ 0.05.

**Results:**

No statistically significant difference was found between group V and group A frameworks regarding passive fit (p-value = 1, OR = 0.5) and time efficiency (*P* = 0.179, Effect size = 0.948).

**Conclusion:**

Within the limitations of this study, it can be concluded that in free end saddle cases, prefabricated CAD\CAM 3-unit implant-supported cobalt chromium screw retained prostheses can achieve an adequate passive fit. However, their fit might be negatively affected in thin ridges and they might require some adjustments.

**Supplementary Information:**

The online version contains supplementary material available at 10.1186/s12903-024-04950-y.

## Background

Kennedy class I and II partial dentures commonly have problems of support, retention, and stability. These problems are solved by the placement of dental implants. This treatment approach offers several advantages, including enhancing patients’ psychological well-being and self-esteem, while ensuring long-term predictable and successful removable or fixed prosthodontic outcomes [1].

Removable implant supported prostheses have the advantages of being hygienic, simple and capable of replacing missing bone and soft tissues, but are characterized by their limited efficiency due to decreased patients’ adaptation and increased maintenance problems [2, 3]. On the other hand, fixed implant supported prostheses (FISP) provide better patient acceptance with reduced bulk and sometimes cost [4]. FISPs are retained either by screws or cement [5]. Screw retained FISPs offer easy and predictable retrieval of the prosthesis, whenever hygienic, reparative, or surgical interventions are needed. Furthermore, less interocclusal space with a minimum of 4 mm could be required for their construction. These advantages overcome their passive fit problem [6].

The growing interest in reducing time between implant placement and final restoration using a functionally and an aesthetically acceptable prosthesis justifies the need for immediate functional loading among dentists [7]. As a result, new treatment ideas were introduced to the dental field, which allow for converting interim prostheses into definitive ones, while fulfilling the prerequisites of immediate loading protocol [8]. Utilizing the advantages of computer aided design/ computer aided manufacturing (CAD/CAM) and the advances in the dental technology, equipment and tools including intra-oral scanners and cone beam computed tomography (CBCT) allow for an accurate transfer of the planned implant positions, depth, and angulations to the patient’s mouth. This might make it possible to construct a metal fixed implant supported prosthesis ahead of placing the implants [9–11]. The fully digital workflow might eliminate the need for impressions and stone casts that could be accompanied with technical errors, shortens the treatment time and reduces the number of visits, while providing the patient with an immediately loaded rigidly splinted definitive prosthesis [12–18]. Metal implant supported fixed prostheses have been successfully fabricated with CAD/CAM in variable span lengths using different materials [19–22]. The rigidly splinted prosthesis lessens the transmission of horizontal forces to the bone, allows for better stress distribution to the implants, prosthesis and supporting structures, splints the implant abutments to improve the retention and the resistance form of the prosthesis and therefore, minimizes screw loosening [23–27]. However, being prefabricated makes them highly liable to problems of passive fit. Hence, in this invitro study we hypothesize that there will be no significant difference between the passive fit of prefabricated and conventionally constructed cobalt chromium CAD/CAM 3-unit implant supported frameworks in Kenndy class I models.

## Materials and methods

This pilot invitro study was reported following the modified consolidated standards for reporting clinical trials (modified CONSORT) statement for invitro studies [28]. The first version of the study protocol was published in protocolexchange on July 2023, with a doi: 10.21203/rs.3.pex-2285/v2 and registration site https://protocolexchange.researchsquare.com/article/pex-2285/v2. The study was carried out on 5 Kennedy class I 3D printed resin models with the first premolar as the last standing abutment. The edentulous ridges of the models were thin wiry, which is a common clinical finding in the mandibular posterior region.

Based on an estimated probability of passive fit for CAD/CAM frameworks constructed on actual implant positions versus those constructed on virtual ones (0.85/0.15), an effect size of 0.7, a power of 80% and an α error = 0.05, when using chi square test, the calculated sample size was 17 frameworks (G*Power version 3.1.9.7, University of Duesseldorf, Germany). Hence, a total of 20 frameworks were constructed, 10 for each group. On each side of the Kennedy class I model 10 frameworks were constructed; 5 for the test, namely the prefabricated frameworks designed based on the virtual implant positions (group V) and 5 for the control group (group A), which were designed based on the actual implant positions. A stone cast model was scanned using an intraoral scanner (Medit i700, Medit Intra-oral scanners). The design of the virtual model was modified using a designing software (Autodesk Meshmixer software, Autodesk, Inc.) so that each side was a mirror image of the other. Additionally, the edentulous areas bilaterally had thin ridges with 1.5 mm thick covering mucosa simulator. Each model was given a code engraved in its base. Using the same software a soft tissue index that served to create an even space of 1.5 mm for the tissue mimic material was designed so that it covered the remaining teeth and the edentulous areas, while extending to the retromolar pads posteriorly to help in confirming the seating of the index. Once the index design was finished, the edentulous areas of the models were trimmed using a cut back tool in the software. The trimmed part was later replaced by tissue mimic material (Zhermack SpA, light body impression material), thereby simulating the resiliency of the oral mucosa in the patient’s mouth. To enhance the retention of the tissue mimic material to the model, retentive holes were cut in the edentulous areas at a depth of 0.5 mm.

**Fig. 1:**
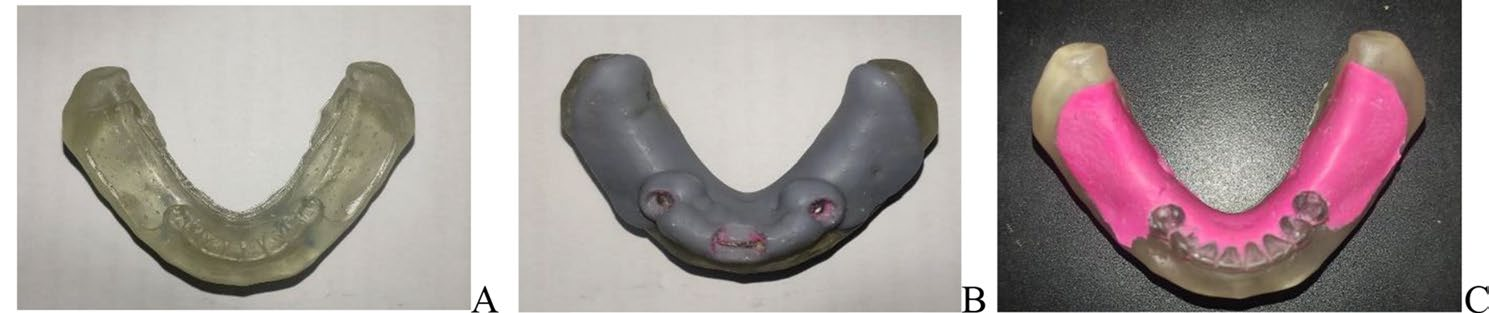
**A**, Printed resin model showing cut back area as spacer and depth cuts as mechanical means of retention for tissue mimic. **B**, Resin printed index used to develop even thickness of 1.5 mm for tissue mimic material. **C**, Resin printed model with tissue mimic attached to it

The modified models were then printed using clear resin (Anycubic High Clear Resin, Anycubic) and a digital light processing (DLP) 3D printer (Anycubic Photon Mono M5s, Anycubic) using the following settings: 0.05 mm layer thickness, exposure time 3 s and zero-degree orientation (Fig. 1A). The index was printed using grey resin (NYCUBIC Grey UV Resin Grey Resin 405 nm for LCD SLA Imprimante 3D, Anycubic) with a 45 degrees orientation using the same printing parameters.

The tissue mimic material was dispensed into the edentulous areas of the printed models and the index was seated until it touched the retromolar pads posteriorly and the incisal and occlusal surfaces of the remaining teeth anteriorly (Fig. 1B, C).

**Fig. 2:**
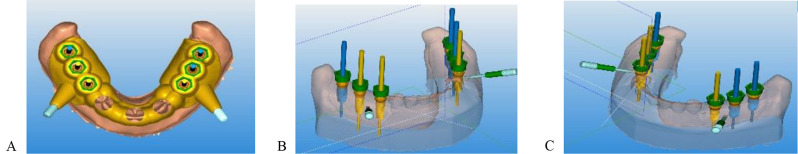
**A**. Design of used surgical guide, **B**. Right side view of fixation pins and planned implant positions, **C**. Left side view of fixation pins and planned implant positions

The model was scanned using the intra-oral scanner and was radiographed using cone beam computed tomography (CBCT) (Planmeca Viso G7, Planmeca). The standard tessellation language (STL) files were imported to a digital designing software (Exocad Dental CAD, Exocad), which was used for the virtual set up of the second premolar, first and second molars bilaterally. The Digital Imaging and Communication in Medicine (DICOM) and the STL files of the models were superimposed using best fit registration technique in an implant planning software (Realguide Software Suite, 3diemme Bioimaging technologies). Once the plan for the implant positions, depth, size and angulations was set, a CAD \CAM surgical guide with a minimum thickness of 4 mm was designed to incorporate metal sleeves (Fig. 2).

The surgical guide was then printed using a clear resin (Anycubic Photon Mono 3D, Anycubic) with a 45 degrees orientation and printing parameters 0.05 mm layer thickness and exposure 1.5 s. Six dummy implants, 3 on each side (3.7 × 10 mm JDental care) were installed by the aid of the guide that was fixed to the model by 2 fixation screws. For Group V, the frameworks were constructed based on virtual implant position and virtual scan bodies that were exported from the implant planning software (Realguide Software Suite, 3diemme Bioimaging technologies) to the designing software (Exocad Dental CAD, Exocad). Accordingly, the designing software converted the virtual scan bodies into virtual multi-unit abutments that were already present in its library. STL files of the framework were segmented from the virtual multiunit abutments and milled before implant placement.

**Fig. 3:**
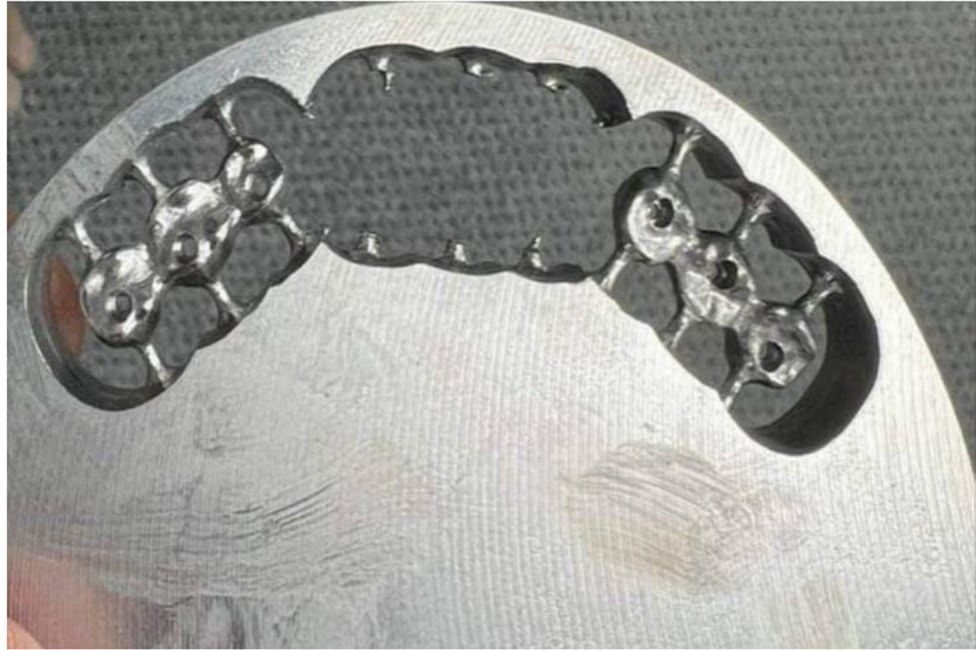
Milled 3-unit implant supported framework from cobalt chromium block

For group A, multiunit abutments (straight conical abutments screwed-in prosthesis, J Dental care) were screwed to the implants by a torque wrench to a torque of 25 Ncm as recommended by the manufacturer. The seating of the multiunit abutments was verified by a periapical digital radiograph (Digora Optime Dexis systems, Soredex). Scan bodies (JD scanbody, J Dental care) were attached to the multiunit abutments for scanning using the intraoral scanner. The latter was used to simulate the clinical situation. The STL files obtained from intraoral scanning were exported to the software for designing the framework. The same procedures followed for constructing the designed frameworks of group V were applied for group A. For identifying the frameworks of both groups, a code was engraved on each framework in the designing phase. Only IR was aware of the codes. The designs of the 20 frameworks, whether of groups A or V were transferred to the CAMing software (Millbox Dental CAM, CIM system), to be milled by the aid of a 5-axis milling machine (Emar ED5x CNC, Milling machine, Emar) using Chromium cobalt blanks (CoCr milling discs, Starbond CoS disc Basic, Scheftner dental alloys, CE 0482) (Fig. 3).

**Fig. 4:**
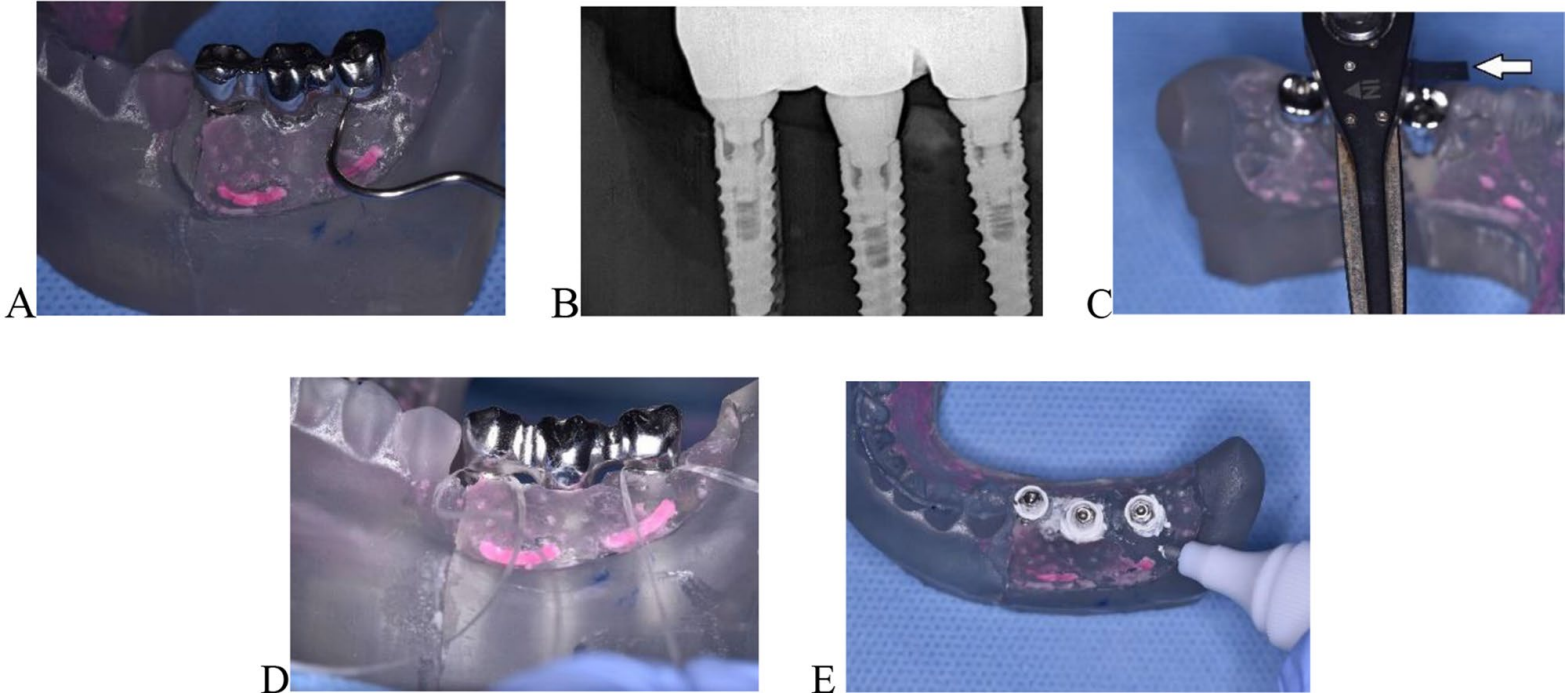
**A**, Tip of probe used for detecting gaps > 60 microns. **B**, Digital peri-apical radiograph to examine gaps between multi-unit abutments and framework. **C**, Flag test done by attaching a plumber’s tape on screwdriver, while pointing to central groove of first premolar and indicating if more than half turn was necessary to achieve passive fit. **D**, Floss slippage test to confirm findings of screw resistance test. **E**, Corrector pen used for checking fit of framework to abutments

They were then screwed to multi-unit abutments attached to implants on the model for outcome assessment. The outcome was assessed by MK, who was blinded to the intervention. According to Araújo et al. [21] passive fit of implant supported prostheses is defined as “a stress-free, simultaneous, circumferential contact at the implant/abutment prosthesis interface before functional loading”. The tissue mimic material was removed at that stage to allow for better visibility during passive fit check. This outcome was reported as a binary outcome and was assessed by 8 different clinical tests as suggested by Abduo el al [29]. These were buccolingual and mesiodistal stability test using alternating pressure, probing test gaps using the diagnostic probe tip (60 microns) (Fig. 4A), the one screw test (Sheffield test) when screwing anterior and posterior abutments, periapical radiograph for macro-gap detection (Fig. 4B) and finally screw resistance test using the flag (Fig. 4C), floss slippage (Oral B unwaxed dental floss, Procter & Gamble) (Fig. 4D) and fit checker tests (Fig. 4E).

All tests were performed in the same sequence for all frameworks to ensure standardization. The results of these tests were compared to the screw resistance test, which was proved to be the second most accurate test after 3D photogrammetry used for measuring the passivity [29].

**Fig. 5:**
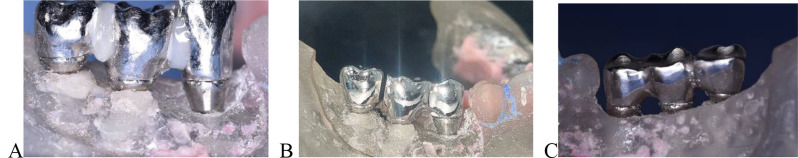
**A**, Sectioned framework. **B**, Assembled framework using flowable composite resin. **C**, Soldered and finished framework

The framework was considered passively fitting when all tests showed passivity. If the framework failed to show passive fit in one or more tests, it was considered not passively fitting. For each framework, a set of new prosthetic screws was used to decrease the possibility of false results due to screw settling. Detailed description for the tests used for examining the passive fit can be seen in Suppl Table 1. Frameworks of both groups that were not passively fitting were tested for interferences in their fitting surfaces by using pressure indicating paste (PIP) (GC Fit Checker Advance, GC corp.) to check for minor discrepancies. Interferences were marked by areas that were denuded from PIP. Using a carbide flame shaped bur with long shank (3 × 6 mm head size, H shape, Harfington) in a low-speed (5000–6000 RPM) straight hand piece these areas were relieved. The framework was checked again for seating and passive fit. The process was repeated until passive fit was achieved. If the passive fit and seating could not be possibly achieved by minor modifications, sectioning of the framework was performed between the 1st and 2nd premolar, between the 2nd premolar and 1st molar or between both. This was done using a carborundum disc (35 × 3 mm, Red, Vision abrasive discs). The sectioned parts were placed on the multiunit abutments and screwed to them and reassembled together using flowable composite (Meta Nexcomp Flowable composite, Meta Biomed) (Fig. 5A). At that time the framework was ready to be sent to the laboratory for soldering using a solder alloy (Kobalt-Chrom-Lot, Ref. 52520, Bego), finishing and polishing (Fig. 5B, C).

Clinical time required starting from the first passive test check till the last modification done in each framework, whether sectioning or relief, was calculated by HM using a stopwatch. Additionally, the laboratory time required for reassembling the sectioned parts was calculated starting from the time at which the framework was sent to the laboratory till the time point when it was delivered back to the operator. The total time, clinical and lab, was also calculated. Data was collected, tabulated, and statistically analyzed. Qualitative data was presented as frequencies and percentages. Numerical data were explored for normality by checking the distribution of data and using tests of normality (Kolmogorov-Smirnov and Shapiro-Wilk tests). Since they showed non-parametric distribution, they were presented as median and range. The comparison between the two groups was done using Mc Nemar’s test for qualitative and Wilcoxon signed rank test for quantitative data. The significance level was set at *P* ≤ 0.05. Statistical analysis was performed with IBM SPSS Statistics for Windows, Version 23.0. Armonk, NY: IBM Corp.

## Results

In group A, 3 frameworks showed instability in mesiodistal and buccolingual directions, while in group V 4 frameworks had this problem and were hence considered not passively fitting. Minor adjustments in the frameworks of group A and sectioning/soldering of group V frameworks achieved the required passive fit. The average time required for sectioning of group V frameworks was 16.66 ± 4.93 min, for soldering 45 ± 6 h with a total average time (clinical and lab) of 45.2 ± 6.07 h. On the other hand, the average clinical time required for adjusting frameworks of group A was 3.33 ± 1.1.52 min.

Studying the effect of the group revealed no significant difference between groups A and V regarding the overall passive fit and number of adjusted frameworks, whether by sectioning/soldering or relief at *P* = 1 and an OR = 0.5, the clinical time at *P* = 0.176, a Cohen’s d effect size = 0.948 and the overall time required for adjustments at *P* = 0.176 and a Cohen’s d effect size = 0.948 (Tables 1 and 2). All tests produced equivalent results to each other and to the screw resistance test.

**Table 1:** Results of McNemar’s test for comparison between the overall passive fit of the two groups on the right and left sides separately and in conjunction. Passively fitting frameworks did not require adjustments

Side	Passive fit	Group V (*n* = 10)		Group A (*n* = 10)		*P*-value	*Effect size (OR)*
		n	%	n	%		
Right	Yes	3	60	3	60	1	0.333
	No	2	40	2	40		
Left	Yes	3	60	4	80	1	0.667
	No	2	40	1	20		
Total	Yes	6	60	7	70	1	0.5
	No	4	40	3	30		

*: Significant at *P* ≤ 0.05, n: number, OR: Odds ratio

**Table 2:** Results of wilcoxon signed-rank test for comparison between clinical and total (clinical and lab) time required for adjustments in group V (sectioning and soldering) and in group A (relief)

Tested outcome	Side	Group V (*n* = 10)		Group A (*n* = 10)		*P*-value	*Effect size (d)*
		Median	Range	Median	Range		
Clinical time in minutes	Right	0	0–20	0	0–3	0.465	0.691
	Left	0	0–15	0	0–5	0.276	1.115
	Total	0	0–20	0	0–5	0.176	0.948
Total time (clinical and lab) in minutes	Right	0	0-2900	0	0–3	0.285	1.089
	Left	0	0-2895	0	0–5	0.461	0.697
	Total	0	0–2900	0	0–5	0.176	0.948

*: Significant at *P* ≤ 0.05, n: number, OR: Odds ratio

## Discussion

The increasing interest in immediate loading of dental implants justifies the need for their splinting to allow for better stress distribution, splint implant abutments to improve retention and resistance form, while minimizing the possibility of screw loosening [22]. Behnaz et al. [18] concluded that implant bodies, cortical and spongy bone experienced less stress because of splinting restorations. These findings suggest that splinting is useful for reducing implant body and cortical bone stresses, especially when a non-axial load as in the oral cavity is applied. Even in posterior regions of partially edentulous patients, where no esthetics is required, several attempts have been performed to immediately or early load implants [26, 27, 30, 31]. This does noy only reduce the number of dental visits, efforts and cost required, but it increases the patient satisfaction regarding functionality and mastication. Schincalgia et al. [30] suggested that immediate loading of implants using fixed partial dentures in the posterior mandible may be considered a treatment option if implants are inserted with an insertion torque ≥ 20 Ncm and ISQ ≥ 60 into nonaugmented bone and loaded with light centric occlusal contact. On the other hand, Borenstein et al. [31] recommended full occlusal contact of early loaded posterior fixed implant supported prostheses. Because of the recent advances in technology, splinting could be made possible not only by using provisional restorations but also by using metal frameworks. However, passive fit of these frameworks might be problematic, especially if fabricated ahead of implant placement.

All the tests that were used in the study were clinical tests aiming to produce clinically relevant results. These tests were chosen because of their simplicity, feasibility, and low cost, whereby they could be done with the simplest tools available in any clinic. The tests were shown to have comparable results to the screw resistance test, which was proven to be the most accurate clinical test [29]. The results of our study revealed no statistically significant difference between frameworks constructed based on virtual and actual implant positions. Both frameworks were constructed by computer aided milling which has been reported to be the most accurate among 2 other techniques, including casting and additive manufacturing (3D printing) [21]. Cobalt chromium frameworks were also reported to be of high quality and great accuracy, with similar surface roughness to titanium, degree of distortion and misfit within an acceptable limit, namely < 150 μm [22]. 

Using prefabricated precision milled superstructures as prosthetic frameworks or bars could drastically reduce material distortion and hence enhance passive fit. Parel and Triplett [32] evaluated the use of a prefabricated bar system for immediately loaded implants placed and restored using the All-on-Four concept over a 24-month period. They reported high success rates and concluded that prefabricated bars for immediately loaded screw retained full arch implant-supported prostheses offer a reliable treatment option for edentulous patients.

Despite the lack of any statistical significance between frameworks of the studied groups, the odds of passive fit of group V was 0.5 times that of group A, suggesting that results are clinically significant. Group A frameworks were constructed based on an actual implant position that was recorded using a highly accurate intra-oral scanner namely Medit i700. Failh and Majeed [33] showed that the design of the finishing line affected the accuracy of the scanner and that the worst trueness and precision value of the Medit i700 is 0.016 ± 0.001 and 0.014 ± 0.002, respectively. This made 7 frameworks of group A fit passively with no adjustments and 3 with only minor adjustments, requiring an average clinical time for adjustment of 3.33 ± 1.1.52 min. On the other hand, 6 frameworks of group V were passively fitting, while the remaining 4 required sectioning and soldering to achieve the required passive fit. The process involved laboratory steps and hence required an average overall time of adjustment of 45.2 ± 6.07 h. Group V frameworks were constructed based on virtual scan bodies and a digital model that was prepared by registering the STL file of the scanned model with the DICOM files of the CBCT. Errors relevant to the imaging, scanning and registration are highly possible [34]. The planned position was transferred then to the cast through a CAD/CAM guide, which could be another source of error. The accuracy of static computer-assisted implant surgery is reported in a clinical study of Kim et al. [35] who stated the three-dimensional linear distance difference between the planned and the placed implant was 0.97 ± 0.37 mm at the cervical and 1.13 ± 0.36 mm at the apical end of the implant. The difference in angle deviation between the planned and the placed implant was 3.42 ± 2.12°. A statistically significant impact has also been reported for the type of implant guide support, implant diameter and implant length. The angulation and depth of the implants, the quality of the bone and supporting mucosa are just a few of the variables that can cause deviations from digital planning. This might explain in a part the clinical significance between the 2 groups, especially when complicated by the thin wiry ridge which called for a deeper placement of the implants and hence contributed to the lack of passive fit with major discrepancies in 4 of the virtual frameworks. Besides, the possible micro-movement of the guide intraoperatively due to the placed gingival simulator over the edentulous ridges, may result in a positional discrepancy between the virtual surgical guide and the printed one during surgery [36]. In an attempt to identify the amount of deviation at each implant in the 4 non-passively fitting frameworks, MS measured them and found that the average linear deviation between the virtual and the actual implant positions was 0.254 mm at the second premolar, 0.38 mm at the first molar and 0.47 mm at the second molar region. The discrepancy was observed to increase as we go posteriorly. This is totally expected, where the support and engagement of the guide by the teeth anteriorly decreased its micro-movements, on contrary to the gingival simulator that covered the thin ridges posteriorly.

The absence of any statistical significance found in our study oppose the results reported in the study of Kheneifer et al. [37]. A total of 10 polyurethane radiopaque anatomic completely edentulous mandibular models had implants placed in the left and right canine and second premolar positions using a 3-dimensionally printed fully guided surgical stent. Twenty implants for each group were loaded using a prefabricated bar designed based on virtual and actual implant positions. The results of the study showed that conventional CAD/CAM milled titanium bars had a better passive and marginal fit than prefabricated CAD/CAM milled titanium bars. However, both had clinically acceptable passive fit ranging from 75.2 to 94.7 μm and definitive marginal fit ranging from 18.7 to 56.3 μm. The difference in the statistical significance of both studies, ours and Kheneifer’s, might be attributed to the variation in the inter-implant distance and the type of guide support. Implant orientation may be incorrectly interpreted by the software algorithms, especially in sizable edentulous areas. The use of IOS for complete dental prostheses is still questionable due to problems related to registering soft tissue dynamics and the absence of reference [38]. The close distance between the placed implants and the presence of some teeth in our study improved the chances of passive fit of the virtual frameworks, thereby creating no significant difference between the 2 studied groups [39]. The ability of digital impression systems to make accurate impressions without generating pressure on the soft tissue suggests that the incorporation of a digital scanner system in making impressions for partially edentulous patients might have resulted in an improved fit if compared to Kheneifer et al. [37, 40, 41].

Possible solutions have been suggested by the authors to overcome problems of framework misfit including customized multi-unit abutments that compensate for discrepancies in depth, angulation and position and provide an unlimited range of variations [42], and the use of stackable metal guides [43], where the metal prosthesis serves as a guide and a final prosthesis simultaneously.

Despite all efforts done to eliminate all variables in this in-vitro study, there are certain limitations. Being in-vitro is a limitation per se, since it does not fully replicate the clinical conditions, including tongue mobility, fogging, presence of saliva, limited mouth opening, presence of impeding structures as opposing teeth and cheek, which could affect the accuracy of the surgical and scanning procedure separately or in conjunction. Additionally, since this was a pilot study investigating an innovative idea, sample size calculation was based on estimates. Hence, a possibility of ß error exists, meaning that the results could have become statistically significant, if the sample size had been calculated. Therefore, further clinical research is still highly recommended to confirm or refute the results of this in vitro study.

## Conclusions

Within the limitations of this study, the following can be concluded that in free end saddle cases, CAD\CAM 3-unit implant-supported prefabricated frameworks can achieve passive fit and offer a possible treatment option in patients indicated for immediate loading.

## Electronic supplementary material

Below is the link to the electronic supplementary material.

Supplementary Material 1

## Data Availability

The datasets used and/or analyzed during the current study are available from the corresponding author on reasonable request.

## Abbreviations

CAD/CAM: Computer Aided Design/ Computer Aided Manufactured
CBCT: Cone Beam Computed Tomography
DICOM: Digital Imaging and Communications in Medicine
FISP: Fixed Implant Supported Prostheses
Group A: Group Actual Implant Position
Group V: Group Virtual Implant Position
PIP: Pressure Indicating Paste
µm: micrometer
mm: millimeter
Ncm: Newton centimeter
STL: Standard Tessellation Language
3D: Three Dimensional
