# Supplementary material for: Passive fit and time efficiency for prefabricated versus conventionally constructed cobalt chromium CAD\CAM 3-unit implant supported frameworks in free end saddle models: a pilot invitro study
Source: BMC Oral Health. 2024 Oct 15;24:1225. doi: 10.1186/s12903-024-04950-y (PMC11481720; doi:10.1186/s12903-024-04950-y)
Supplement: Supplementary file 1 — Supplementary Material 1 [file 12903_2024_4950_MOESM1_ESM.docx]

**TABLES (Suppl)**

Table Suppl 1. The required tests for passive fit evaluation

| Test | Procedure |
| --- | --- |
| 1- Stability test (Alternating finger pressure test)^44^  mesiodistally  2- buccolingually | Alternating pressure was applied across the framework to check its stability in a buccolingual and mesiodistal direction. The tests aimed to evaluate the frameworks for presence of any lifting or rocking. |
| 3- Visual examination (probing)^45^ | Using a conventional examination probe with a tip 60 microns in diameter, the interface between the framework and the multi-unit abutments was checked for any gaps. A gap that accommodated the tip of the probe is ≥60 microns. This was used in combination with the one screw test performed at the terminal abutments. |
| 4- The one screw test (Sheffield test anteriorly and posteriorly)^46^ | The screw of the framework was tightened to anterior abutment at 15 Ncm as recommended by the manufacturer. Gaps observed at the 2 other abutments were checked by a periapical radiograph and directly by the probe tip as it ran all around the abutment prosthesis interface**.** The same test was repeated when tightening the framework to the posterior abutment. |
| 5-Periapical radiograph^47^ | Digital periapical radiographs were taken using wireless sensors and long cone paralleling technique with XCP Rinn film holder to ensure an accurate vision of any gaps or discrepancies at the framework abutment interface. The image was visualized on a software (Digora)^^[[1]](#footnote-1)^^**.**This was used in combination with the one screw test that was performed on the terminal abutments. |
| 6- Screw resistance test (Flag test)^46^ | In which a tape was placed around the shaft of the screw driver in the form of a flag. This flag served as a marker for the assessor to identify the turns a screw had made when attempting to achieve maximum screw seating according to the manufacturer’s guidelines. Screws were tightened one by one, starting with the implant in the midline until initial resistance between the head of the screw and the framework was encountered. A maximum of one-half turn (180 degrees) was then allowed to completely seat the screw and achieve a torque of 15 Ncm. A misfit existed if more than a half turn was needed to achieve the desired screw seating and torque measurement. |
| 7-Floss slippage test^48^ | Unwaxed dental floss 12 microns in thickness was applied at the framework abutment interface. For frameworks with gaps at the abutment-framework interface > 12 microns, the floss slipped. This test was used in conjunction with the screw resistance test, when only the middle screw was tightened and the floss applied at the two terminal abutments. |
| 8-Fit checker^49^ | A pressure indicating paste was applied as a thin layer on the abutment platform. The presence of disclosing media at the mating surface of the framework abutment interface, anteriorly, centrally or posteriorly indicated misfit. This test was used to complement the screw resistance test. |

1. [↑](#footnote-ref-1)
